# A Hybrid Metamaterial for Broadband, High-Performance Sound Absorption via Helmholtz Resonators Incorporating Porous Media

**DOI:** 10.3390/ma19173651

**Published:** 2026-08-27

**Authors:** Zhifu Zhang, Yuan Liao, Rui Xue, Naikui Chen, Yizhe Huang, Jingru Li

**Affiliations:** 1 School of Mechanical and Electrical Engineering, Hainan University, Haikou 570228, China; zfzhang@hainanu.edu.cn (Z.Z.); 24220854060014@hainanu.edu.cn (Y.L.); 25220855090002@hainanu.edu.cn (N.C.); 2 Hubei Key Laboratory of Modern Manufacturing Quality Engineering, School of Mechanical Engineering, Hubei University of Technology, Wuhan 430068, China

**Keywords:** hybrid metamaterial, broadband sound absorption, multi-cavity material, Helmholtz resonator, JCA model

## Abstract

**Highlights:**

- A hybrid metamaterial couples HR resonance and PR porous dissipation on one surface.
- Zero-pole analysis separates low-frequency HR resonance from broadband porous dissipation.
- Peak optimization broadens α ≥ 0.7 absorption to 360–563 Hz.
- The design helps overcome the low-frequency/broadband absorption trade-off.
- The zero-pole method guides mechanism-based absorber tuning.
- The nine-cell material shows manufacturable broadband low-frequency absorption.

**Abstract:**

Finite-thickness acoustic absorbers often face a trade-off between low-frequency resonance and broadband dissipation. The porous material (PR) provides stable mid-to-high-frequency losses through viscous and thermal effects, whereas Helmholtz resonators (HRs) offer compact low-frequency resonance but usually operate over narrow bands. Accordingly, an HR–PR hybrid metamaterial absorber is developed by coupling a central air neck and a sealed back cavity with a surrounding porous layer described by the Johnson–Champoux–Allard (JCA) rigid-frame equivalent-fluid model. A surface impedance model is formulated for the HR branch, the PR branch, and their area-weighted parallel admittance and is validated against COMSOL 6.4 unit-cell simulations. Complex-frequency reflection zero-pole analysis separates the HR-dominated localized resonance from the broadband dissipative contribution of the porous branch. Nine-unit cells with different back-cavity volumes are then arranged into a 3 × 3 composite material to distribute the HR resonances across the target low-frequency range. Using a peak placement optimization strategy to identify a broad continuous absorption band satisfying a sound absorption coefficient (*α*) ≥ 0.7, the equivalent model predicts an absorption band of 360–563 Hz with a bandwidth of 203 Hz. Air impedance tube measurements show the same overall absorption trend as the theoretical and numerical results, supporting the effectiveness of the optimized HR–PR hybrid metamaterial absorber.

## 1. Introduction

Low-frequency noise is difficult to suppress in architectural acoustics, equipment cabins, and duct systems because of its long wavelength, slow propagation attenuation, and strong structural penetration. Improving low-frequency absorption within a limited installation space cannot rely simply on increasing the thickness of porous materials (PRs); instead, compact designs must coordinate localized resonance, impedance matching, and material dissipation [1,2,3]. Porous media dissipate sound mainly through viscous friction and thermal exchange between pore air and the solid frame. They are structurally simple, easy to fabricate, and effective in the mid-to-high-frequency range; however, at low frequencies, when the material thickness is much smaller than the acoustic wavelength, the velocity and temperature gradients inside the pores are insufficiently developed, which limits the absorption efficiency [4,5,6]. By contrast, HRs use the coupling between the inertia of the air column in the neck and the acoustic compliance of the back cavity to form localized resonance, inducing strong local pressure and velocity oscillations at subwavelength scales and thereby compensating for the low-frequency deficiency of porous materials [7,8,9]. Nevertheless, the response of a single HR is typically confined to a narrow frequency band, and its off-resonance absorption is limited. Therefore, broadband low-frequency absorbers should be designed by considering porous dissipation, HR localized resonance, and their impedance-matching relationship simultaneously.

Locally resonant acoustic materials have evolved from low-frequency band-gap control to thin-layer effective parameter modulation and, more recently, broadband absorption regulation [10,11,12]. Early studies used subwavelength resonant units to overcome the dependence of conventional phononic crystals on periodic-length scales. Wang et al. [11] developed a broadband absorber based on parallel square Helmholtz resonators (HRs) and showed that localized resonant units can tune low-frequency absorption within compact geometries. Membrane-type materials and acoustic metasurfaces subsequently extended localized resonance to thin low-frequency control; for example, Zhang et al. [13] used digitally controlled piezoelectric metamaterials to improve low-frequency and high-efficiency sound absorption, providing a recent active route for thin resonant materials. Multimodal coupling and critical coupling have since been introduced to broaden absorption bandwidths. Nie et al. [14] used a hybrid Helmholtz–helical metamaterial for broadband-targeted noise suppression, while Romero-Garcia et al. [15] demonstrated broadband absorption through critically coupled subwavelength resonators. Equivalent-fluid models of porous materials describe viscous–thermal losses in pore air through dynamic density and dynamic bulk modulus, allowing porous branches to be incorporated into composite absorbers in impedance form. Recent porous material studies, for instance, the soft porous absorber discussed in [16], support the use of viscous–thermal loss descriptions for porous branches; together with recent HR–porous absorber studies [17,18,19], this provides the basis for Johnson–Champoux–Allard (JCA) parameterized modeling in the present work. Thus, the central challenge of broadband low-frequency absorption is no longer to obtain a single high absorption peak, but to maintain high absorption over a continuous band through resonance placement, internal dissipation, and resistive compensation between peaks [20,21,22].

Recent studies have related the porous acoustic response to particle- and pore-scale characteristics, including granular molecular sieves with different pore and pellet sizes, hollow-sphere composites, and polyurethane foams characterized through airflow resistivity, impedance tube measurements, and inverse JCA identification [23,24,25]. Other studies have used graded or anisotropic porous media, porous–resonant hybrids, and optimized microperforated–porous configurations to broaden absorption or improve low-frequency performance within constrained thicknesses [26,27]. Work on angle-dependent absorption and transmission loss prediction further shows that normal-incidence absorption, diffuse-field response, and acoustic–mechanical transmission require different validation strategies [28,29]. These studies indicate that porous acoustic performance is governed jointly by pore-scale transport, effective constitutive parameters, resonant coupling, and the measurement configuration. At the modeling level, recent elastoacoustic formulations have treated acoustic media as biphasic systems involving the interaction between an acoustic phase and a deformable mechanical phase. Such formulations explicitly couple acoustic pressure with solid-frame motion, allowing frame-borne waves, elastic deformation, and acoustic–mechanical transmission to be analyzed [30]. In contrast, the present study adopts the rigid-frame JCA equivalent-fluid model, in which the porous skeleton is assumed to be acoustically rigid and stationary. Accordingly, frame elasticity, solid-borne wave propagation, and thermoelastic coupling are not considered. The present work therefore focuses on the rigid-frame JCA surface impedance coupling between HR resonance and PR dissipation under normal incidence, while detailed pore material design, diffuse-field behavior, and application-level transmission loss validation remain outside the present scope.

Within this established research context, the present study makes three methodological contributions. First, the HR localized resonance branch and the PR porous dissipation branch are placed on the same incident surface, and the unit-cell surface impedance is formulated using an area-weighted admittance parallel relationship rather than a simple serial or empirical superposition of the two absorption mechanisms. Second, complex-frequency reflection zero-pole analysis is introduced to distinguish the reflection zeros close to the real-frequency axis, which are associated with low-frequency HR localized resonance, from the zeros farther from the real axis, which correspond to the broadband dissipative background generated by the JCA branch [31]. This provides a mechanistic basis for interpreting peak shifts, bandwidth variation, and impedance matching. Third, a peak placement optimization method is developed for the nine-cell composite material to identify a broad continuous absorption band. Here, α denotes the normal-incidence sound absorption coefficient. In this study, α ≥ 0.7 is adopted as an engineering criterion for evaluating the continuous absorption band [32,33,34]. The optimized cavity volumes are further converted into a manufacturable minimum-height specimen using a 100 mm effective working area, 2 mm cell gaps, and a uniform back-cavity thickness.

Following these contributions, an equivalent surface impedance model is first established for the HR–PR unit cell, clarifying the geometric relationships among the neck, back cavity, porous layer, and incident area, and defining the PR parameters used in the theoretical calculation. COMSOL simulations are then used to validate the absorption responses of the pure HR, pure PR, and composite HR–PR unit cells, thereby identifying the roles of low-frequency localized resonance and mid-to-high-frequency porous dissipation [35,36,37]. A 3 × 3 nine-cell composite material is subsequently constructed, in which different back-cavity volumes distribute the HR branch resonances. The agreement between theory and simulation is evaluated using continuous bandwidth, inter-peak valley levels, and the overall curve trend rather than a single dominant peak. Finally, a peak placement optimization algorithm is designed to identify a feasible distribution of HR peak frequencies that broadens the continuous absorption band for α ≥ 0.7, yielding the optimized target frequencies, back-cavity volumes, and equivalent cavity heights, and the absorption trend is verified experimentally using an air impedance tube.

## 2. Theoretical Methodology

**Figure 1:**
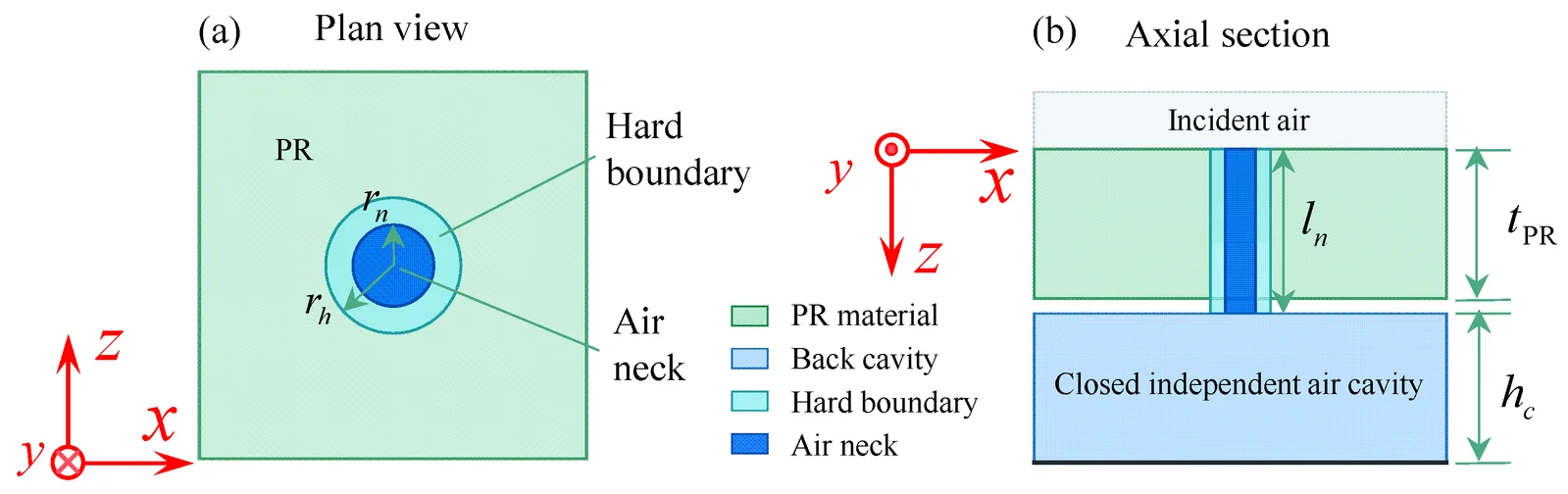
Geometry of the HR–PR unit cell: (**a**) plan view; (**b**) sectional view.

The proposed HR–PR unit cell consists of a central air neck, a rigid sealed back cavity, and a porous layer surrounding the neck. The central neck and back cavity constitute the low-frequency HR resonant branch. The main geometric parameters are the unit-cell cross-sectional area *S*_cell_; neck radius *r_n_*; neck length *l_n_*; outer radius of the rigid boundary *r_h_*; rigid acoustic boundary thickness *t_h_*, which is *r_h_*-*r_n_*; back-cavity height *h_c_*; back-cavity volume *V_c_*; and porous material thickness *t*_PR_. The unit-cell geometry is illustrated in Figure 1. The central cylinder is the air neck, the annular region outside the neck is the rigid acoustic boundary, the remaining region is the PR porous layer, and the rear part is a rigidly sealed air cavity.

Under normal incidence, the acoustic energy entering the structural surface follows two transfer paths. In the first path, sound enters the sealed back cavity through the central neck; the air column in the neck oscillates and couples with the compressible air in the cavity, forming a typical mass spring localized resonance. In the second path, sound enters the PR porous region around the neck, where velocity and temperature gradients are generated near the complex frame surfaces, and acoustic energy is converted into viscous and thermal losses. The incident sound field can be regarded as the superposition of incident and reflected plane waves [38,39]:

(1) $$p(z)=p_{i}e^{-jk_{0}z}+p_{r}e^{jk_{0}z}$$

where *p_i_* and *p_r_* are the amplitudes of the incident and reflected waves, respectively, and *k*_0_ is the wavenumber in air. Because the pressure and particle velocity in a plane wave differ by the characteristic impedance of air, *Z*_0_ = *ρ*_0_*c*_0_, *ρ*_0_ is the ambient air density, *c*_0_ is the sound speed of air, and the corresponding normal particle velocity is:

(2) $$u(z)=\frac{1}{Z_{0}}\left(p_{i}e^{-jk_{0}z}-p_{r}e^{jk_{0}z}\right)$$

The absorption capacity of the material is governed by the matching between its surface impedance and the characteristic impedance of air. At the structural surface *z* = 0,

(3) $$Z_{s}=\frac{p(0)}{u(0)}$$

Substituting the expressions for pressure and velocity gives the surface reflection coefficient as:

(4) $$R=\frac{p_{r}}{p_{i}}=\frac{Z_{s}-Z_{0}}{Z_{s}+Z_{0}}$$

Therefore, the sound absorption coefficient is written as:

(5) $$\alpha =1-{\left|R\right|}^{2}=1-{\left|\frac{Z_{s}-Z_{0}}{Z_{s}+Z_{0}}\right|}^{2}$$

For the low-frequency HR branch [40,41], the neck mainly provides acoustic inertia, whereas the sealed back cavity provides acoustic compliance. When the imaginary parts of the neck inertia and cavity compliance cancel each other, the HR branch resonates at low frequency. *L_e_* denotes the effective neck length including end correction [42]. The volume velocity impedance of the neck is expressed as:

(6) $$Z_{n}=R_{n}+j\omega \frac{\rho_{0}L_{e}}{S_{n}},\;L_{e}=l_{n}+1.7r_{n},\;R_{n}=\frac{L_{e}}{\pi r_{n}^{3}}\sqrt{2\rho_{0}\eta \omega }$$

where *R_n_* denotes the viscous loss inside the neck, the second term represents the inertial effect of the air column in the neck, *η* is the dynamic viscosity of air, *ω* = 2π*f* is the angular frequency corresponding to the excitation frequency *f*, and the neck cross-sectional area is *S_n_* = π$r_{n}^{2}$. The volume velocity impedance of the sealed back cavity is:

(7) $$Z_{c}=-j\frac{\rho_{0}c_{0}^{2}}{\omega V_{c}}$$

This term has a negative imaginary part and represents the acoustic compliance generated by compression of the air in the back cavity. Thus, the total impedance of the HR branch is:

(8) $$Z_{\mathrm{HR}}=Z_{n}+Z_{c}$$

When the imaginary parts of the neck inertia and back-cavity compliance cancel each other, the HR branch reaches low-frequency resonance. The approximate HR resonance frequency is therefore:

(9) $$f_{0}=\frac{c_{0}}{2\pi }\sqrt{\frac{S_{n}}{V_{c}L_{e}}}$$

Consequently, when the neck geometry is fixed, increasing the back-cavity volume *V_c_* lowers the resonance frequency, whereas increasing the neck cross-sectional area *S_n_* raises it.

The PR branch describes the viscous and thermal dissipation in the porous layer surrounding the neck. The JCA model treats the pore air as an equivalent fluid with frequency-dependent density and bulk modulus [43,44]. Accordingly, the solid skeleton is assumed to be acoustically rigid and motionless; its elastic deformation and frame-borne wave propagation are not explicitly modeled. The viscous–thermal interaction between the pore air and the rigid pore walls is retained through the JCA effective density, effective bulk modulus, and thermal characteristic length. No separate thermoelastic response of the solid skeleton is considered. The dynamic effective density is:

(10) $$\rho_{\mathrm{eq}}(\omega )=\frac{\rho_{0}\alpha_{\infty }}{\phi }\left[1+\frac{\sigma \phi }{j\omega \rho_{0}\alpha_{\infty }}\sqrt{1+j\frac{4\alpha_{\infty }^{2}\eta \rho_{0}\omega }{\sigma^{2}\Lambda^{2}\phi^{2}}}\right]$$

where *ϕ* is the porosity, *σ* is the flow resistivity, *α*_∞_ is the high-frequency tortuosity, and Λ is the viscous characteristic length. These parameters provide a physically meaningful connection between pore structure, transport behavior, and acoustic response. Specifically, *ϕ* represents the acoustically accessible pore volume; *σ* characterizes viscous pressure loss through the connected pore network; *α*_∞_ accounts for the increased inertial path associated with tortuous pore pathways; and Λ describes the near-wall scale governing viscous boundary layer interaction. Thus, pore size, pore connectivity, and pore morphology influence the acoustic response through their effects on these transport parameters. The dynamic effective bulk modulus is:

(11) $$K_{\mathrm{eq}}(\omega )=\frac{\gamma P_{0}/\phi }{\gamma -(\gamma -1){\left[1+\frac{8\eta }{j\omega \rho_{0}Pr{\Lambda^{\prime }}^{2}}\sqrt{1+j\frac{\omega \rho_{0}Pr{\Lambda^{\prime }}^{2}}{16\eta }}\right]}^{-1}}$$

where Λ′ is the thermal characteristic length, *P_r_* is the Prandtl number, *γ* is the specific heat ratio of air, and *P*_0_ is the ambient static pressure. Λ′ governs the thermal exchange between the oscillating pore air and the effectively rigid pore walls. The characteristic impedance and propagation constant of the porous layer can be further obtained from *ρ*_eq_ and *K*_eq_ as:

(12) $$Z_{\mathrm{PR}}^{c}=\sqrt{\rho_{\mathrm{eq}}K_{\mathrm{eq}}},\;k_{\mathrm{PR}}=\omega \sqrt{\frac{\rho_{\mathrm{eq}}}{K_{\mathrm{eq}}}}$$

Because the PR layer in this study is backed by a rigid termination, its surface impedance can be written as:

(13) $$Z_{\mathrm{PR}}=-jZ_{\mathrm{PR}}^{c}\cot(k_{\mathrm{PR}}t_{\mathrm{PR}})$$

The HR and PR branches are subjected to the same surface pressure on the incident plane of the unit cell; therefore, admittances rather than impedances should be connected in parallel. After considering the area occupied by the neck and rigid boundary, the total unit-cell surface admittance is:

(14) $$Y_{\mathrm{cell}}=\frac{1}{S_{\mathrm{cell}}Z_{\mathrm{HR}}}+\frac{\xi_{\mathrm{PR},\mathrm{cell}}}{Z_{\mathrm{PR}}},\;\xi_{\mathrm{PR},\mathrm{cell}}=\frac{S_{\mathrm{cell}}-\pi r_{h}^{2}}{S_{\mathrm{cell}}}$$

where *Y*_cell_ represents the area-normalized admittance contributed by the central HR branch, and $\xi_{\mathrm{PR},\mathrm{cell}}$ represents the admittance of the PR branch weighted by its effective area. The equivalent unit-cell surface impedance is:

(15) $$Z_{\mathrm{cell}}=\frac{1}{Y_{\mathrm{cell}}}$$

Substituting *Z*_cell_ into the reflection coefficient equation yields the theoretical absorption coefficient of the HR–PR unit cell:

(16) $$\alpha_{\mathrm{cell}}=1-{\left|\frac{Z_{\mathrm{cell}}-Z_{0}}{Z_{\mathrm{cell}}+Z_{0}}\right|}^{2}$$

To quantify the agreement among the theoretical, numerical, and experimental absorption curves, the root-mean-square error (RMSE) between two data sets *A* and *B* is defined as:

(17) $${\mathrm{RMSE}}_{A,B}=\sqrt{\frac{1}{N}\sum_{i=1}^{N}{\left[\alpha_{A}(f_{i})-\alpha_{B}(f_{i})\right]}^{2}}$$

where *N* is the number of frequency points in the selected comparison range, and *α_A_*(*f_i_*) and *α_B_*(*f_i_*) are the absorption coefficients of data sets *A* and *B* at frequency *f_i_*, respectively. Before calculating the RMSE, the curves are interpolated onto a common frequency.

To further identify the physical origin of the absorption peaks, the normal-incidence reflection coefficient is extended from the real-frequency axis to the complex-frequency plane. The harmonic time convention used throughout this analysis is:

(18) p(r,t)=Rep^(r,ω˜)exp(iω˜t)

Here, *r* = (*x*, *y*, *z*) denotes the spatial position vector, and $\hat{p}$ is the complex acoustic pressure amplitude. The complex frequency and the corresponding complex angular frequency are defined as:

(19) $$\tilde{f}=f_{r}+if_{i},\;\tilde{\omega }=2\pi \tilde{f},$$

where *f*_r_ and *f*_i_ are the real and imaginary frequency components, respectively. The HR impedance, PR effective density, PR effective bulk modulus, porous layer impedance, and unit-cell surface impedance are evaluated at the same complex angular frequency. For the unit-cell analysis, the equivalent unit-cell surface impedance *Z*_cell_ is substituted into the reflection coefficient expression. Thus, the complex-frequency reflection coefficient is:

(20) $$R(\tilde{f})=\frac{Z_{\mathrm{cell}}(\tilde{f})-Z_{0}}{Z_{\mathrm{cell}}(\tilde{f})+Z_{0}}$$

The complex-frequency reflection map is evaluated on a two-dimensional (*f*_r_, *f*_i_) grid and displayed as:

(21) $$M(f_{r},f_{i})=\log_{10}{\left|R(\tilde{f})\right|}^{2}$$

The reflection zeros and poles are calculated from the numerator and denominator of the complex-frequency reflection coefficient. A reflection zero satisfies $Z_{\mathrm{cell}}(\tilde{f})-Z_{0}=0$, whereas a reflection pole satisfies $Z_{\mathrm{cell}}(\tilde{f})+Z_{0}=0$. For reflection zeros, the following two equations are solved:

(22) ReZcell(ω˜)−Z0=0, ImZcell(ω˜)−Z0= 0.

For reflection poles, the following two equations are solved:

(23) ReZcell(ω˜)+Z0=0, ImZcell(ω˜)+Z0= 0.

The above equations are solved using a two-variable damped Newton iteration. Defining the unknown vector $x={\left[f_{r},f_{i}\right]}^{T}$ and denoting the real-imaginary residual vector for a zero or pole by F(x), the numerical update is:

(24) $$J\left(x^{\left(n\right)}\right)\Delta x^{\left(n\right)}=-F\left(x^{\left(n\right)}\right),\;x^{\left(n+1\right)}=x^{\left(n\right)}\lambda \Delta x^{\left(n\right)},\;0\;<\;\lambda \;\leq \;1.$$

where J is the numerically evaluated Jacobian matrix, F is the residual vector for either a zero or a pole, and *λ* is a damping factor selected to reduce the residual norm. The initial value of *f*_r_ is set to *f*_0_, whereas the initial value of *f*_i_ is selected from the local complex-frequency response. The iteration is allowed to proceed for a maximum of 60 steps. If a full Newton step increases the residual norm, *λ* is successively reduced, with a lower limit of 10^−4^. Convergence is accepted when the residual norm is below 10^−6^.

### 2.1. Theoretical and Numerical Validation

**Figure 2:**
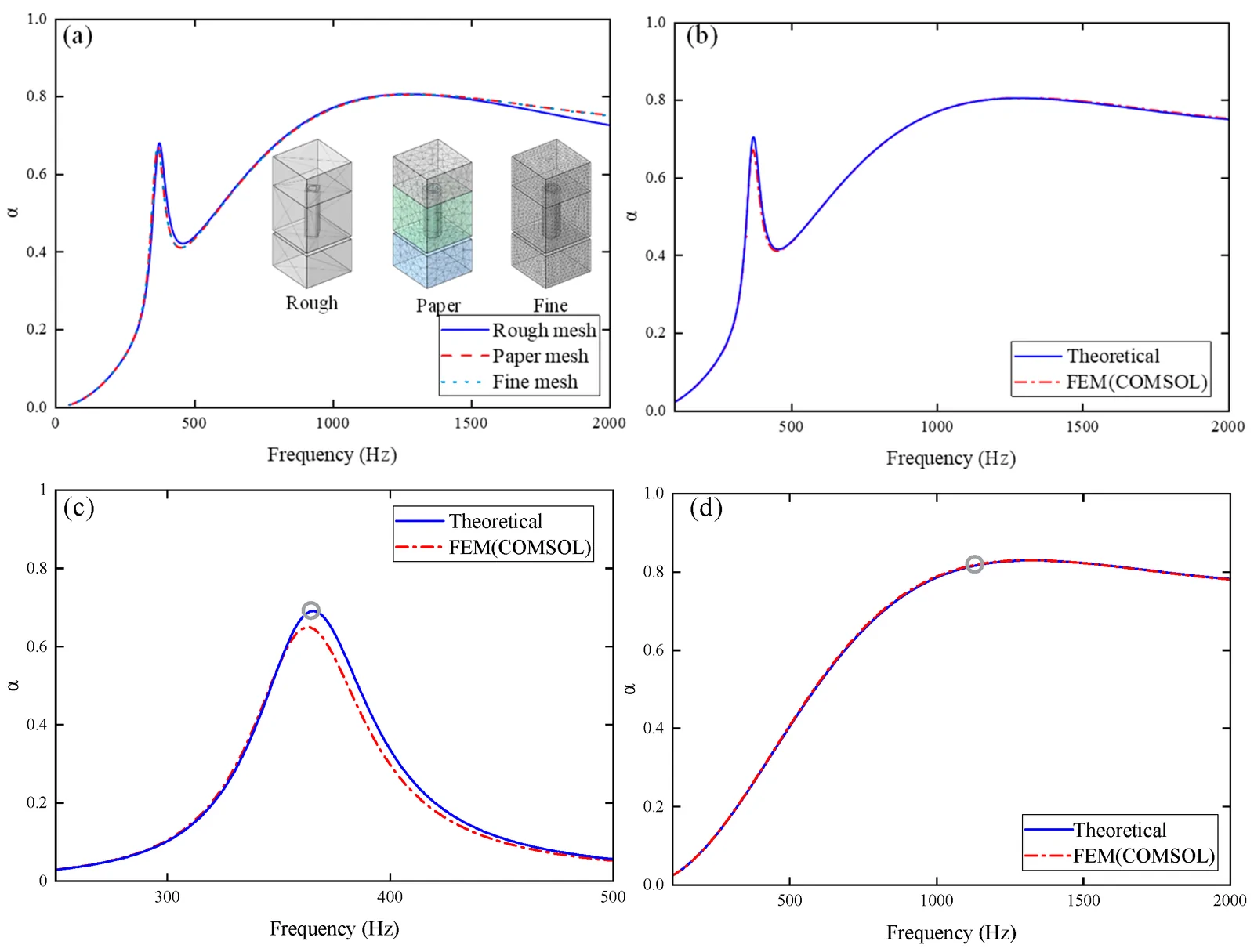
Mesh convergence verification and theoretical numerical validation of the HR, PR, and HR–PR unit cells: (**a**) mesh convergence results; (**b**) HR–PR composite unit cell; (**c**) pure HR unit cell; (**d**) pure PR porous layer.

The proposed HR–PR material is modeled in COMSOL Multiphysics 6.4 using the three-dimensional Pressure Acoustics, Frequency Domain interface. The computational domain comprised the exterior air region, the circular neck, the sealed back cavity, and the porous region. The exterior air domain height is 25 mm, and the acoustic field variable is the complex pressure *p*. A plane-wave Port boundary with an incident pressure amplitude of 1 Pa is assigned at the incident plane. All exterior structural walls and the rigid rear termination are assigned Sound Hard boundaries, while pressure and normal-velocity continuity are imposed at the internal acoustic interfaces. The complex reflection coefficient *R* is obtained from the reflected wave amplitude at the Port, and the normal-incidence absorption coefficient is calculated as α = 1 − |*R*|^2^. The circular neck is modeled using the Narrow Region Acoustics Model to include viscous–thermal boundary layer losses. The porous domain is described by the rigid-frame JCA equivalent-fluid model. Accordingly, the polyurethane skeleton is assumed to be acoustically rigid and motionless; elastic deformation, frame-borne waves, and solid thermoelastic coupling are not included. The model is solved from 50 to 2000 Hz with a frequency increment of 1 Hz. As shown in Figure 2a, a mesh convergence study is performed by progressively refining the neck and porous interface regions. Because further refinement produced no changes in the relevant absorption peak positions or amplitudes, a free-tetrahedral mesh is adopted to balance numerical accuracy and computational efficiency, with global element sizes of 1.12–5 mm and local element sizes of 0.8–2 mm in the neck and porous interface regions. According to the acoustic FEM criterion *h*_max_ < *λ*_min_/10 = *c*_0_/(10*f*_max_), *f*_max_ = 2000 Hz and *c*_0_ = 340 m/s give *h*_max_ < 17 mm; therefore, the adopted maximum element size of 5 mm satisfies this requirement [45]. The final mesh contains approximately 6584 tetrahedral elements, 1738 triangular boundary elements, and 1535 mesh vertices. The frequency domain system is solved using a fully coupled direct solver with the MUMPS backend.

The geometric parameters used in this section are *a_x_* = *a_y_* = 30 mm, *r_n_* = 3 mm, *l_n_* = 30 mm, *t_h_* = 2 mm, and *h_c_* = 20 mm. The PR layer is made of polyurethane foam, and its effective JCA parameters are adopted directly from the experimental characterization of the same material reported by Wang et al. [46]. In that study, the porosity and airflow resistivity were measured using a sealed volume change method and a steady-flow pressure drop method, respectively. The high-frequency tortuosity, viscous characteristic length, and thermal characteristic length were subsequently identified by globally optimizing the JCA model against the measured normal-incidence absorption coefficient obtained using a two-microphone impedance tube with rigid-back termination. PR material thickness, bulk density and material parameters are *t*_PR_ = 28 mm, *ρ*_PR_ = 80 kg/m^3^, *ɸ* = 0.844, *σ* = 64,300 Pa·s/m^2^, *α*_∞_ = 1.34, Λ = 19.9 um, and Λ′ = 46.4 um. The air parameters are *ρ*_0_ = 1.21 kg/m^3^, *c*_0_ = 340 m/s, *η* = 1.84 × 10^−5^ Pa·s, *γ* = 1.40, *P*_0_ = 101,325 Pa, and *P_r_* = 0.71. Figure 2 compares the pure HR, pure porous layer, and HR–PR composite cases to validate the localized HR resonance, porous dissipation, and their area-weighted parallel coupling [47,48].

Figure 2b compares the theoretical and numerical absorption responses of the HR–PR composite unit cell. The composite retains the low-frequency localized resonance introduced by the HR branch, while the PR branch provides broadband viscous–thermal dissipation at mid-to-high frequencies. Over 50–2000 Hz, the theoretical and numerical results agree well, with an RMSE of 0.0056. Figure 2c shows that the pure HR unit cell reproduces the low-frequency resonance position well, with theoretical and numerical peak frequencies of 366 and 364 Hz, respectively; its RMSE over 50–2000 Hz is 0.0085. The remaining difference in peak magnitude is mainly associated with the simplified treatment of viscous loss and the nonuniform velocity distribution near the neck ends in the equivalent model. Figure 2d shows excellent agreement for the pure PR layer, with an RMSE of 0.0019 over the common 100–2000 Hz range. Its broad absorption response confirms that the PR layer dissipation is governed by distributed viscous–thermal effects rather than a single localized resonance. Compared with the pure PR layer, the composite response shifts slightly towards lower frequencies, indicating that the HR branch modifies the total surface impedance through its parallel admittance even outside the main HR resonance region.

To further connect the two dominant absorption mechanisms of the composite unit cell, the complex-frequency reflection map of the HR–PR unit cell is first evaluated in the low-frequency HR resonance region and the high-frequency PR dissipation region [49]. For the low-frequency HR–PR unit-cell map, the real-frequency search interval is [350, 450] Hz, and the imaginary-frequency search interval is [−80, 80] Hz. The map is evaluated using 420 × 320 grid points, corresponding to an approximately 0.36 Hz spacing in the real-frequency direction and approximately 0.50 Hz spacing in the imaginary-frequency direction. The local minima and maxima identified on this grid are used only as initial estimates for the subsequent root calculation; they are not treated as exact mathematical zeros or poles. The complex square-root terms in the PR expressions are evaluated using the principal complex square-root branch at each complex-frequency point. The same branch convention is applied to the PR effective density, effective bulk modulus, characteristic impedance, and propagation constant. On the positive real-frequency axis, this branch gives the passive response used by the real-frequency PR calculation. This description reports the actual implementation and does not introduce an additional branch continuation procedure.

**Figure 3:**
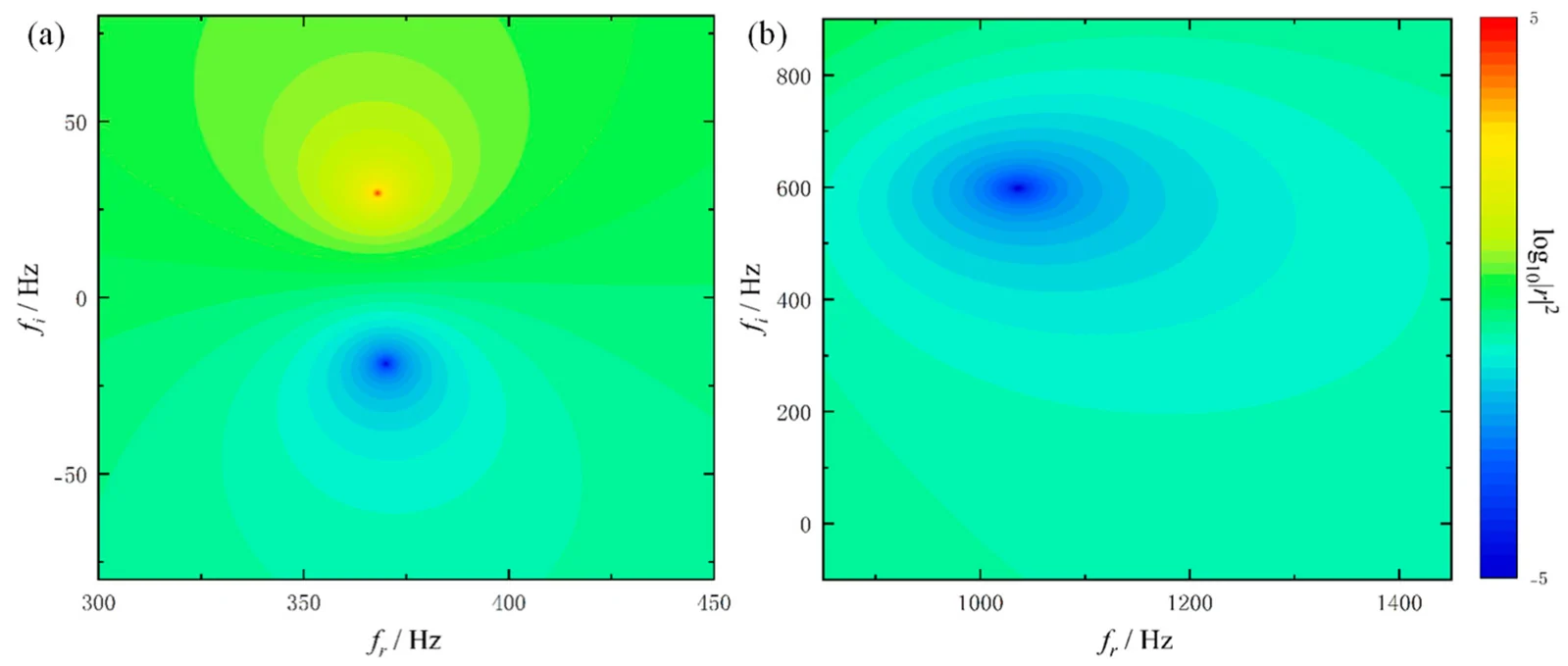
Complex-frequency plane of the unit-cell reflection coefficient: (**a**) low-frequency HR resonance; (**b**) high-frequency PR dissipation.

Figure 3 then compares the resulting zero-pole distributions with the real-frequency absorption response. In Figure 3a, the zero is located close to the real-frequency axis, and its real part is close to the real-frequency absorption peak, indicating that this peak is mainly generated by localized resonance between the neck inertia and back-cavity compliance. An adjacent pole also appears near the zero, showing that the low-frequency peak has a strong narrowband resonant character. Figure 3b shows that the dominant high-frequency zero is farther from the real-frequency axis and does not form a nearby zero-pole pair similar to the low-frequency HR peak. This indicates that the PR branch mainly regulates surface impedance continuously through viscous–thermal dissipation, producing a broad dissipative platform in the real-frequency absorption curve. For the representative unit cell defined above, the damped Newton procedure gives a reflection zero at 370.028568 − 18.725880*i* and a neighboring pole at 368.022751 + 29.638586*i*. The corresponding residual norms are 1.50 × 10^−8^ and 9.29 × 10^−9^, respectively, both below the convergence criterion of 10^−6^. These results quantitatively confirm that the low-frequency absorption peak is associated with a closely spaced zero-pole pair near the real-frequency axis. Accordingly, the pure HR unit cell forms a narrowband low-frequency resonance peak, whereas the pure PR layer forms a broadband mid-to-high-frequency dissipation platform. After coupling the two branches, the low-frequency HR peak is retained, and the mid-to-high-frequency absorption is provided by the PR branch.

### 2.2. Parametric Analysis

**Figure 4:**
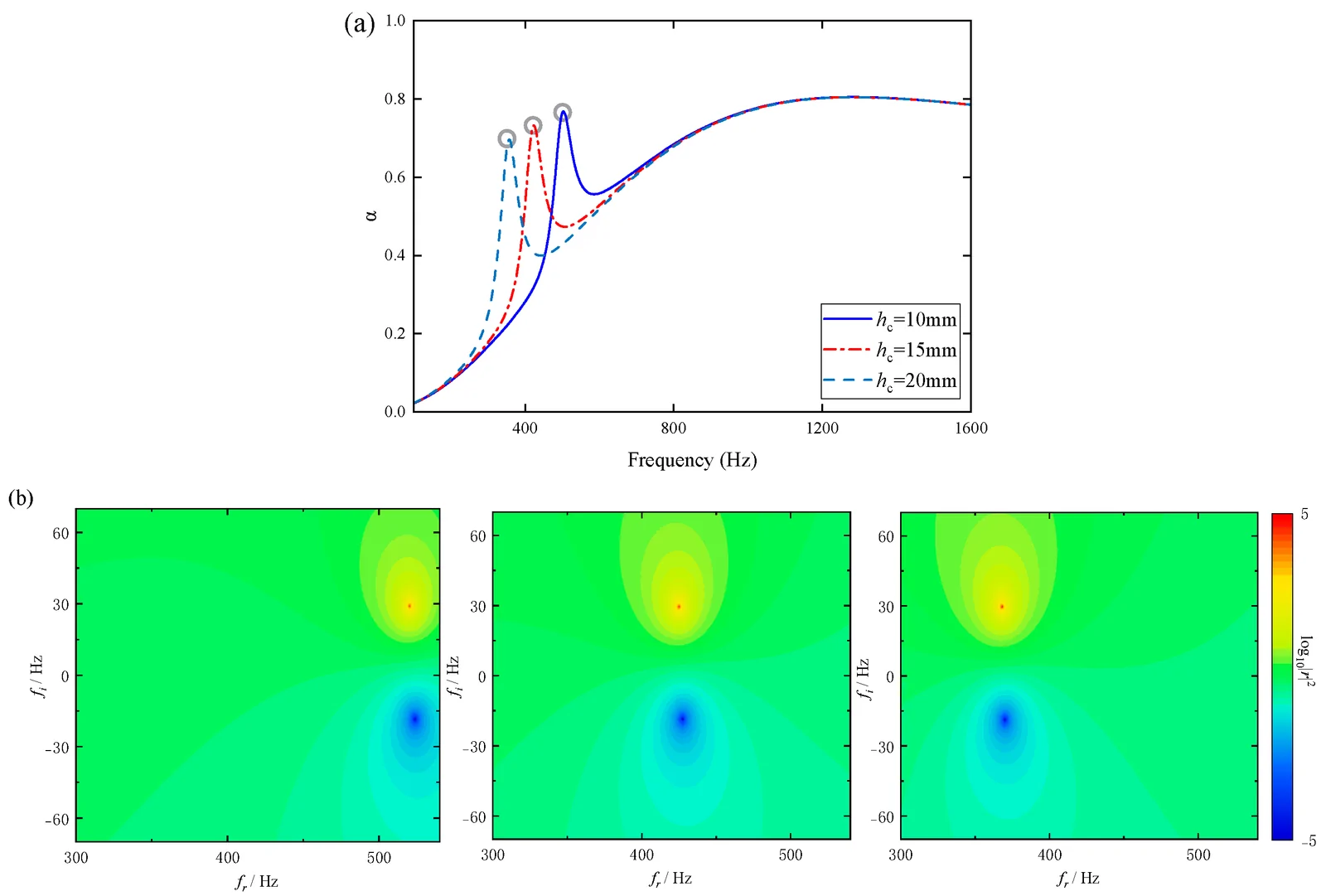
Influence of back-cavity height *h_c_* on the HR–PR unit-cell absorption response: (**a**) absorption coefficient; (**b**) zero-pole map.

After validating the theoretical model using unit-cell simulations, a single parameter analysis is performed for the geometric parameters of the HR branch. First, *r_n_* = 3 mm, *l_n_* = 30 mm, the PR parameters, and the back-cavity cross-sectional area of 30 × 30 mm are kept unchanged, and back-cavity heights of *h_c_* = 10, 15, and 20 mm are selected. The corresponding absorption coefficients and zero-pole maps are shown in Figure 4 [50].

Figure 4a shows that, when the back-cavity cross-sectional area is fixed, increasing *h_c_* shifts the resonance frequency toward lower frequencies according to *f*_0_ ∝ $V_{c}^{-1/2}$ and *V_c_* = S_c_*h_c_. This occurs because the neck air mass and the cavity air spring cancel their reactance at a lower frequency. The high-frequency position and the overall shape of the curve vary only slightly since the porous branch keeps the same material thickness, area ratio, and PR parameters. As shown in Figure 4b, increasing the back-cavity height causes a pronounced decrease in the real parts of the zeros and poles, while the magnitudes of the imaginary parts remain almost unchanged. This nearly horizontal movement suggests that, in the present model, the back-cavity height mainly shifts the resonance frequency and has little effect on the effective loss. The back-cavity volume can therefore be used as a tuning variable in the composite design. Different *V_i_* values may first be selected to place several zeros within the target low-frequency range, after which the remaining parameters can be adjusted to improve the connection between adjacent peaks.

**Figure 5:**
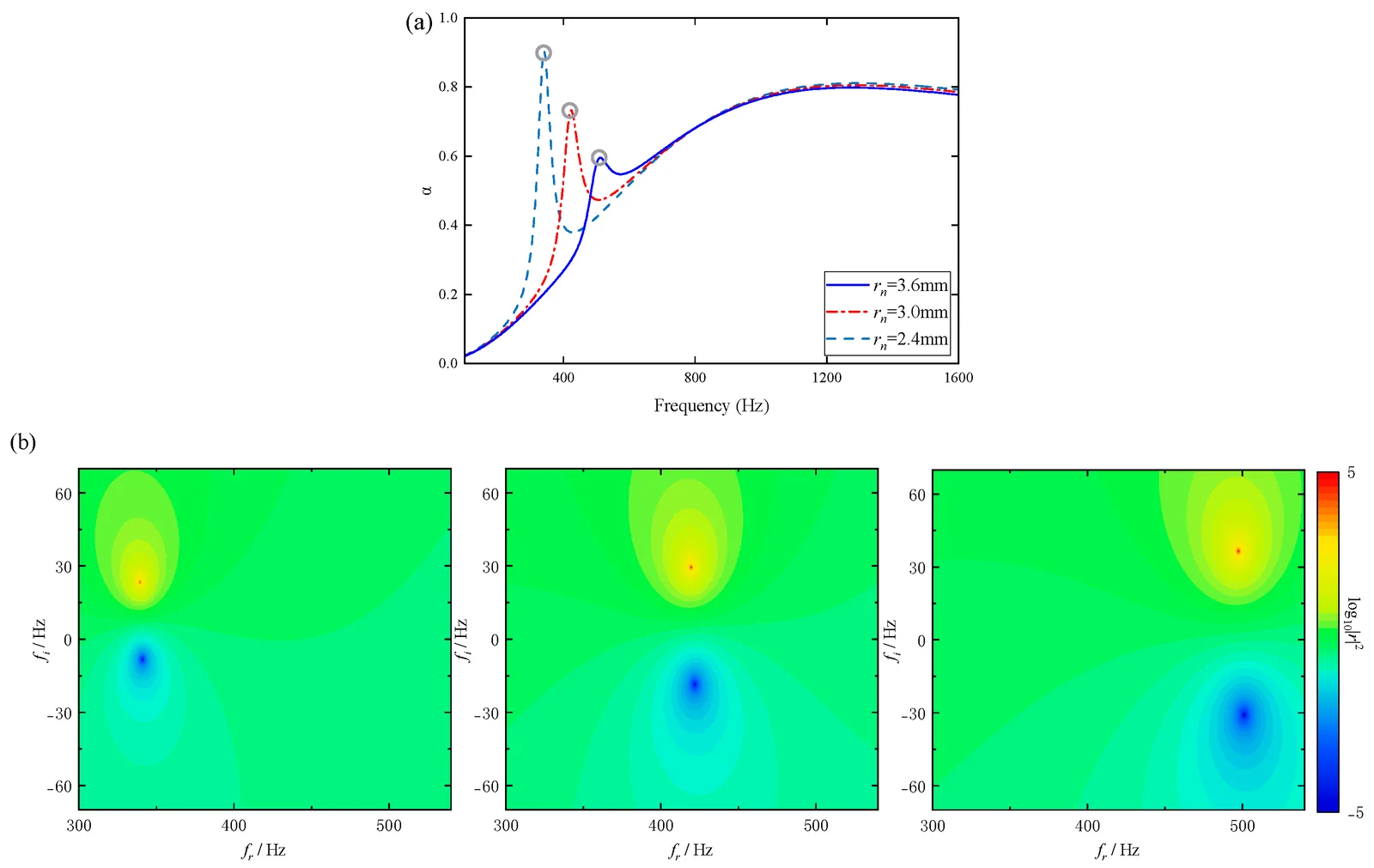
Influence of neck radius *r_n_* on the HR–PR unit-cell absorption response: (**a**) absorption coefficient; (**b**) zero-pole map.

Next, *h_c_* = 15 mm, *l_n_* = 30 mm, and the PR parameters are kept unchanged, and neck radii of *r_n_* = 2.4, 3.0, and 3.6 mm are selected with a uniform increment of 0.6 mm. The absorption coefficients and zero-pole maps calculated from the theoretical model are shown in Figure 5.

The theoretical analysis shows that the low-frequency resonance frequency of the material approximately satisfies:

(25) $$f_{0}=\frac{c_{0}}{2\pi }\sqrt{\frac{\pi r_{n}^{2}}{V_{c}(l_{n}+1.7r_{n})}}$$

Figure 5a shows that increasing *r_n_* shifts the low-frequency resonance peak toward higher frequencies. This rightward shift originates from the increase in the neck cross-sectional area: a larger neck area reduces the inertial impedance corresponding to a given volume velocity, allowing the neck mass and cavity compliance to reach balance at a higher frequency. However, the peak value does not increase synchronously with the neck radius, indicating that impedance matching deteriorates in addition to the peak position shift. This behavior is associated with the change in HR branch admittance and viscous loss caused by the larger neck area, as well as the reduction in the effective incident area of the PR branch. Figure 5b reveals this difference: as *r_n_* increases, the zeros and poles not only move toward higher frequencies but also become farther from the real-frequency axis. The increasing distance between the zero and the real-frequency axis corresponds to the reduction in the real-frequency peak value, while the change in the pole imaginary part reflects modified radiation coupling. Thus, the neck radius is a strong parameter that simultaneously affects peak position, peak magnitude, and bandwidth.

**Figure 6:**
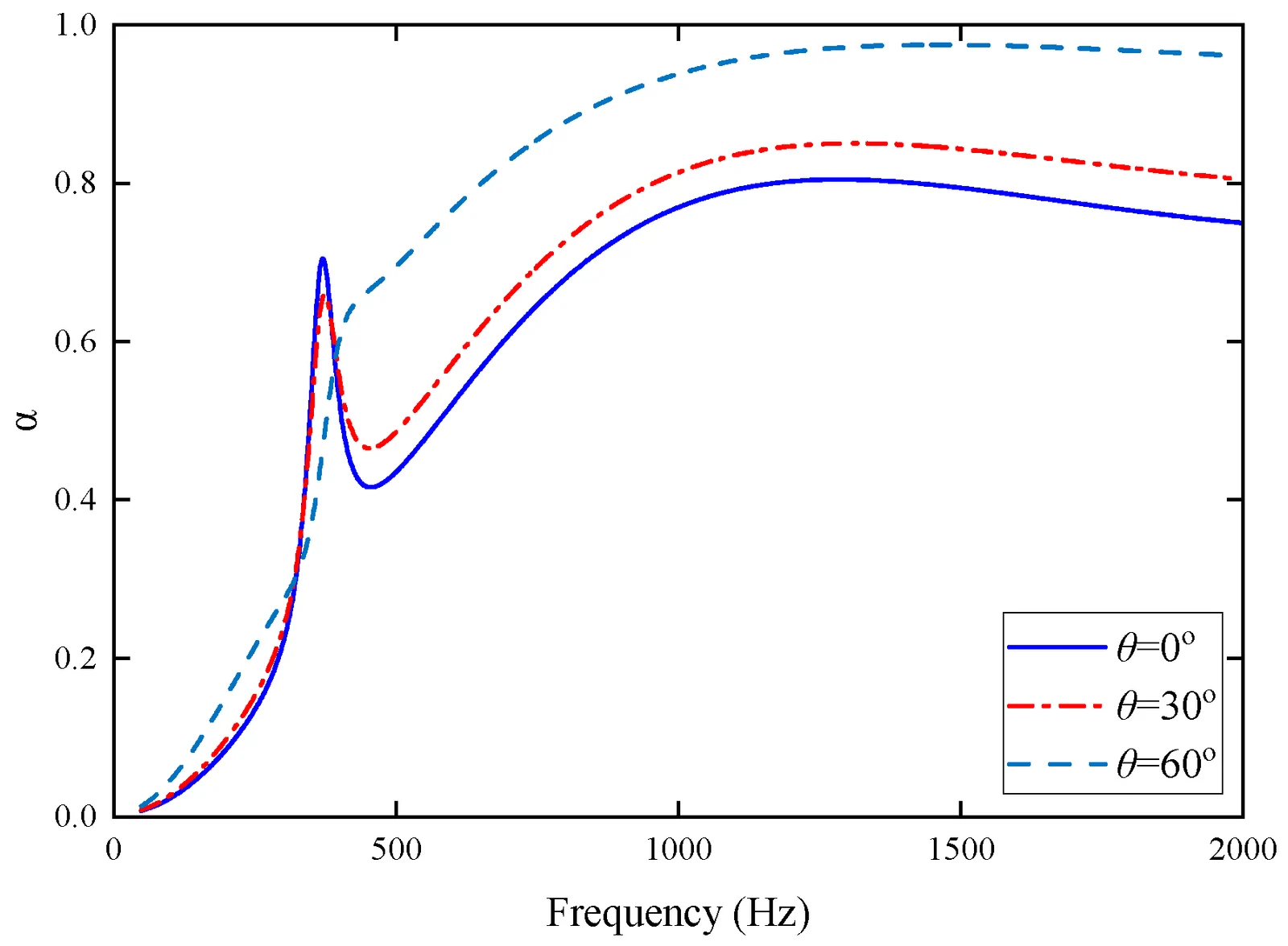
Theoretical sound absorption coefficient of the HR–PR unit cell at incidence angles of 0°, 30° and 60°.

Finally, the influence of the incidence angle on the response of an individual HR–PR unit cell is investigated. The back-cavity height is fixed at *h_c_* = 20 mm. The neck radius and neck length are fixed at *r_n_* = 3 mm and *l_n_* = 30 mm, respectively. The PR layer thickness and all PR parameters are kept unchanged. The theoretical absorption responses at incidence angles of 0°, 30° and 60° are compared in Figure 6.

For an obliquely incident plane wave, the absorption coefficient of the single-unit-cell theoretical model is calculated as:

(26) $$\alpha_{\theta }(f)=1-{\left|\frac{Z_{\mathrm{cell}}(f)\cos\theta -Z_{0}}{Z_{\mathrm{cell}}(f)\cos\theta +Z_{0}}\right|}^{2}$$

Here, *θ* is the incidence angle, and *Z*_cell_(*f*) is the surface impedance calculated from the same area-weighted parallel-admittance model defined above. In the present angle-dependent analysis, the HR–PR branch impedances are retained in the same forms as those used for normal incidence. The incidence-angle dependence is introduced through the normal component of the incident-wave impedance, *Z*_air,*n*_ = *Z*_0_/cos*θ*, which is equivalent to the cos*θ* terms in the above expression. Thus, *Z*_air,*n*_ increases from Z_0_ at 0° to 1.155Z_0_ at 30° and 2*Z*_0_ at 60°. As the incidence angle increases from 0° to 60°, the low-frequency HR feature remains evident at 0° and 30°, with the peak changing only slightly from 372 Hz with *α* = 0.704 to 375 Hz with *α* = 0.659. At 60°, this low-frequency peak is no longer distinct, while the full-range absorption reaches a maximum of 0.975 at approximately 1463 Hz. Therefore, the enhanced higher-frequency absorption results from the combined effects of the altered impedance-matching condition and the angle-dependent viscous–thermal dissipation of the PR layer, rather than from a direct shift of the original HR resonance toward higher frequencies.

In summary, the back-cavity volume causes nearly horizontal zero-pole migration and is therefore suitable for determining the target low-frequency peaks of the composite material. The neck radius causes oblique migration and is more suitable for adjusting critical coupling and the valley values between adjacent peaks after the peak positions have been initially determined. Accordingly, the composite material can first use *V_i_* to design the target low-frequency peaks, and then use a uniform or locally varied *r_n_* to tune impedance matching and inter-peak valleys. In addition, the single-unit-cell angular analysis shows that increasing the incidence angle increases the air-side target impedance from *Z*_0_ to *Z*_0_/cos*θ*, thereby progressively weakening the impedance matching of the low-frequency HR resonance while enhancing the higher-frequency response through the combined effects of air-side matching and PR viscous–thermal dissipation. Therefore, the incidence-angle dependence should be considered when the composite material is intended for oblique sound incidence.

## 3. Composite Material Design and Optimization

### 3.1. Theoretical and Simulation Comparison for the Composite Material

The nine-cell material adopts a fixed 3 × 3 grid. All unit cells have the same neck radius, neck length, and incident area, and the low-frequency HR peaks are staggered only by changing the back-cavity volumes. The total admittance of the composite material can be written as the area-weighted sum of the HR and PR branches:

(27) $$Y_{\mathrm{array}}(f)=\sum_{i=1}^{9}\frac{1}{S_{0}Z_{\mathrm{HR},i}(f)}+\frac{\xi_{\mathrm{PR},\mathrm{array}}}{Z_{\mathrm{PR}}(f)},\;Z_{\mathrm{array}}=\frac{1}{Y_{\mathrm{array}}}$$

When the neck geometry is fixed, the target resonance frequency of the *i*-th branch still satisfies:

(28) $$f_{0,i}=\frac{c_{0}}{2\pi }\sqrt{\frac{S_{n}}{V_{i}L_{e}}}$$

**Figure 7:**
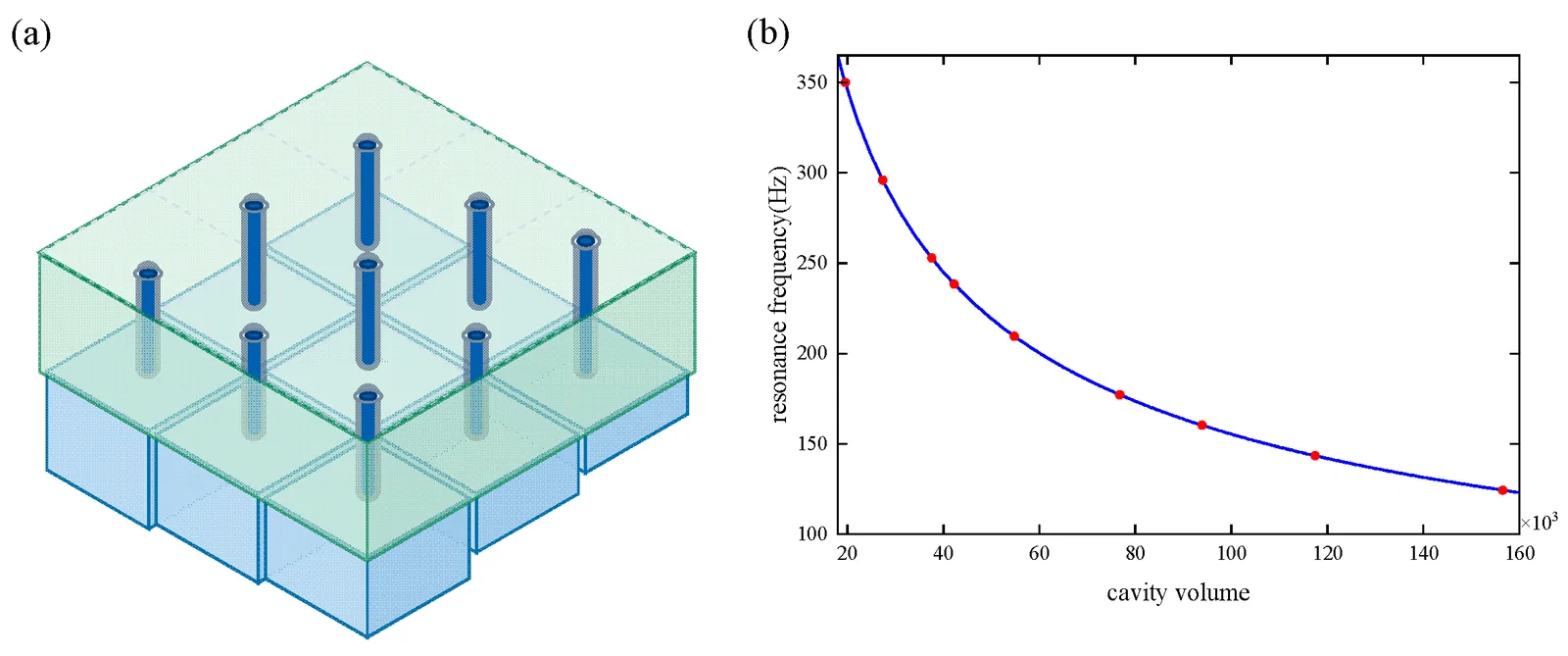
Nine-cell composite material and back-cavity volume–resonance frequency relationship: (**a**) composite geometry; (**b**) resonance frequency as a function of back-cavity volume.

Therefore, the back-cavity volume can be used as a direct design variable for multi-peak placement. Figure 7 shows the geometry of the nine-cell composite material and the inverse-square-root relationship between cavity volume and target frequency.

A 3 × 3 nine-cell composite material is designed in this section, as illustrated in Figure 7a. The external cross-section of the composite material is 100 × 100 mm, and the nine unit cells are arranged in a fixed 3 × 3 grid. The nine cells have the same neck radius, neck length, and incident area, whereas different back-cavity volumes are used to stagger the HR peaks over the low-frequency range. Together with the dissipative background provided by the PR branch, these staggered resonances form broadband absorption. In Figure 7b, the volume–frequency curve decreases monotonically and nonlinearly. In the small-volume region, the same volume variation causes a larger frequency change; in the large-volume region, the sensitivity of frequency to volume gradually decreases. Therefore, equal spacing in volume does not produce equal spacing in frequency. The red dots in Figure 7b denote the back-cavity volumes used in this study.

**Figure 8:**
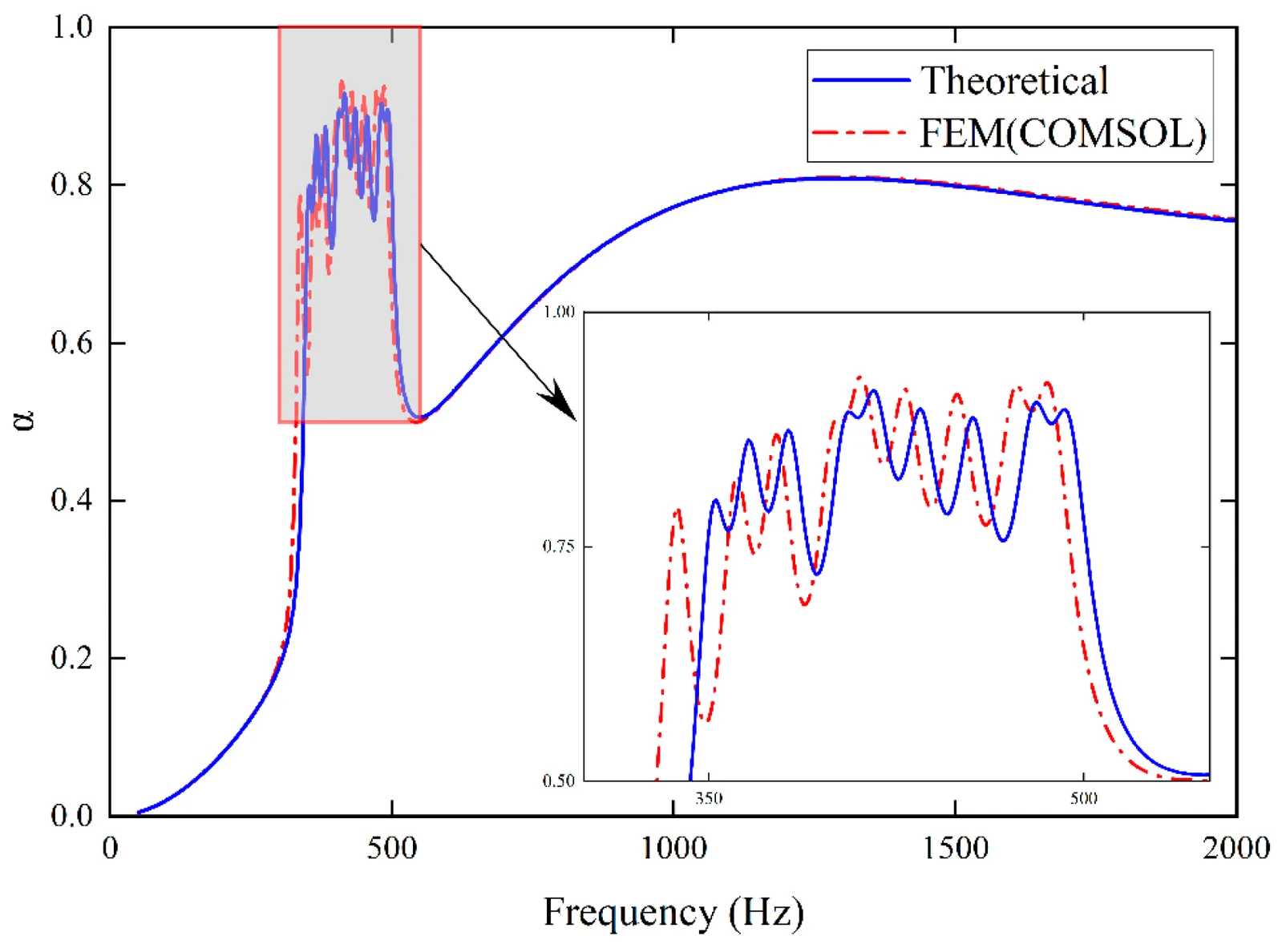
Comparison between theoretical and COMSOL absorption coefficients of the nine-cell composite material.

Figure 8 compares the theoretical and COMSOL-simulated absorption responses of the nine-cell composite structure. Over the full frequency range of 50–2000 Hz, the RMSE between the two curves is 0.0378. In the low-frequency region, both responses exhibit the expected multi-peak absorption behavior associated with the nine HR branches, although the COMSOL curve shows sharper variations in the valleys between adjacent peaks. These differences are mainly attributed to the three-dimensional sound field, the velocity distribution near the neck ends, air boundary effects, and finite-size coupling between neighboring cells. Overall, the results confirm that multiple back-cavity volumes transform the single narrow resonance into an overlapping multi-peak absorption response.

### 3.2. Broadband Absorption Optimization of the Composite Material

To obtain a wider high-absorption band, this study uses the continuous frequency interval satisfying *α* ≥ 0.7 as the optimization target under the prescribed ranges of nine back-cavity volumes and structural constraints. To illustrate the influence of different volume distributions on the connection between peaks, five candidate distributions are first compared, including the initial nine-volume distribution, uniform-volume spacing, frequency spacing, low-frequency dense spacing, and high-frequency dense spacing. The corresponding equivalent straight-cavity heights are listed in Table 1.

The above candidate schemes reveal the main design trends. To avoid manually selecting a distribution, the volume distribution is further formulated as an optimization problem, where *f_i_* is the target resonance frequency of the *i*-th branch. The corresponding back-cavity volume and optimization objective are:

(29) $$V_{i}=\frac{S_{n}}{L_{e}}{\left(\frac{c_{0}}{2\pi f_{i}}\right)}^{2},\;\max_{f_{1},\ldots ,f_{9}}\;W_{0.7}=f_{b}-f_{a}$$

F_0_ denotes the initial candidate set generated from the five preset distributions; Δ*f* = {25, 15, 10, 7, 5, 3, 2, 1, 0.5} Hz denotes the step size set; *α*_th_ = 0.7 is the absorption threshold; *W*_0.7_ = *f_b_* − *f_a_* is the longest continuous bandwidth satisfying *α*(*f*) ≥ α_th_; and *f_i_*, *V_i_*, and *h_i_* are the target frequency, back-cavity volume, and equivalent straight-cavity height of the *i*-th HR branch, respectively. Here, [*f_a_*, *f_b_*] is within [250, 750] Hz, and all frequency points in this interval satisfy *α*(*f*) ≥ *α*_th_. To maintain the low-frequency absorption target, the start of the continuous band is constrained by *f_a_* ≤ 360 Hz. The target peak frequencies are constrained by 280 ≤ *f*_1_ ≤ ⋯ ≤ *f*_9_ ≤ 700 Hz, with at least 2 Hz spacing between adjacent peaks, and the equivalent straight-cavity height is limited to 5.4–30 mm. The procedure starts from the initial distribution, the uniform distribution, and several initial points covering the low-frequency range. At each step, only feasible schemes that increase *W*_0.7_ are retained until the step size converges.

To ensure reproducibility of the bandwidth search, the procedure is formulated as a continuous-bandwidth peak placement optimization algorithm with the criterion *α* ≥ *α*_th_. The target peak frequencies of the nine HR branches are taken as the search variables. The objective is to identify a feasible peak frequency distribution that produces a broad continuous interval satisfying *α* ≥ *α*_th_, subject to the cavity volume constraint that makes each candidate peak set geometrically feasible. Rather than choosing from five predefined volume distributions, the algorithm iteratively links candidate initialization, frequency-to-volume conversion, bandwidth evaluation, and stepwise peak perturbation. The full procedure is summarized in Algorithm 1.
**Algorithm 1:** Optimization algorithm for broadband absorption designInput*r_n_*, *l_n_*_,_ F_0_, Δ*f*, *α*_th_, *h_i_*.Output*W*_0.7_, *f_i_*, *V_i_* and *h_i_*.1Generate F_0_ from five candidate distributions.2For each F in F_0_.3Convert *f_i_* to *V_i_* and *h_i_*.4Check *h_i_* and peak spacing.5Compute *α*(*f*) from the area-weighted parallel admittance.6Find the longest *α*(*f*) ≥ *α*_th_ band.7Update the best solution if *f_a_* ≤ 360 Hz and *W*_0.7_ increases.8For each step in Δ*f*.9Generate trials by perturbation, shift, and scaling.10Accept feasible trials with larger *W*_0.7_.11Stop when no improvement appears.12Return the best *f_i_*, *V_i_*, *h_i_*, and *W*_0.7_.

The optimized scheme obtained by the algorithm covers 360–563 Hz, yielding a continuous bandwidth of 203 Hz under the *α* ≥ *α*_th_ criterion. The corresponding target frequencies and equivalent heights are listed in Table 2.

**Figure 9:**
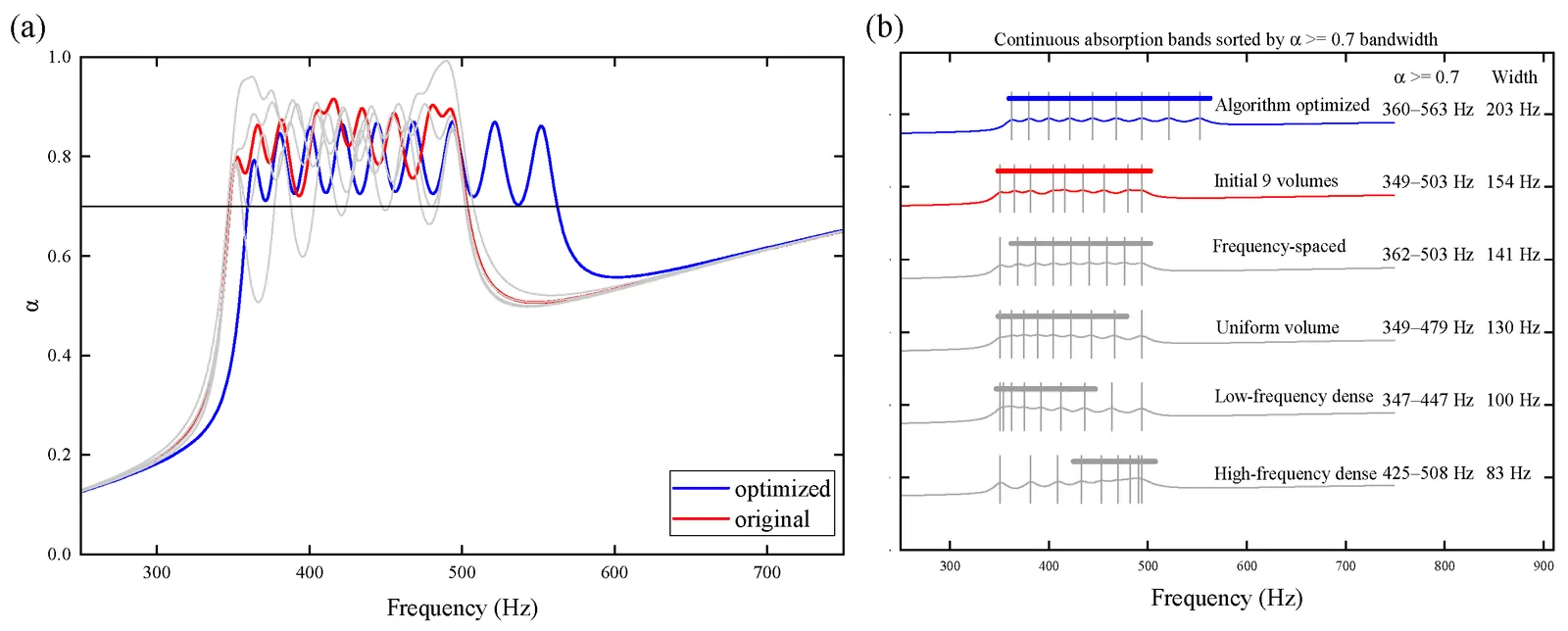
Comparison of continuous *α* ≥ 0.7 absorption bandwidths for preset volume distributions and the optimized scheme: (**a**) composite absorption curves; (**b**) frequency ranges ranked by continuous bandwidth.

Therefore, under the *α* ≥ 0.7 criterion, the optimized scheme increases the low-frequency continuous bandwidth from 154 Hz for the best-performing preset distribution to 203 Hz. This indicates that subsequent design should not rely solely on preset volume distribution patterns; instead, the continuous bandwidth should be solved directly as the objective function. The corresponding visualization is shown in Figure 9.

Figure 9a compares the composite absorption curves of the initial nine-volume scheme and the algorithm-optimized scheme, with other candidate schemes shown in gray. In the initial scheme, the absorption coefficient first reaches the threshold at 349 Hz and drops below it at about 503 Hz. After optimization, the onset moves slightly upward to 360 Hz, while the upper limit extends to 563 Hz. The optimization therefore does not act as a simple frequency shift. Instead, it redistributes the nine absorption peaks, trading a small loss at the low-frequency edge for broader high-frequency coverage and a smoother connection between adjacent peaks. Figure 9b shows the longest continuous frequency band with *α* ≥ 0.7 for each scheme, together with the corresponding target peak positions. The line segment reflects whether the valleys between neighboring peaks remain above the threshold, while the grey markers identify the HR branches contributing to the band. In the optimized design, the target peaks span 360–563 Hz, with their spacing progressively adjusted across frequency to balance the low-frequency onset, inter-peak overlap, and high-frequency extension.

## 4. Experimental Validation of Broadband Sound Absorption

**Figure 10:**
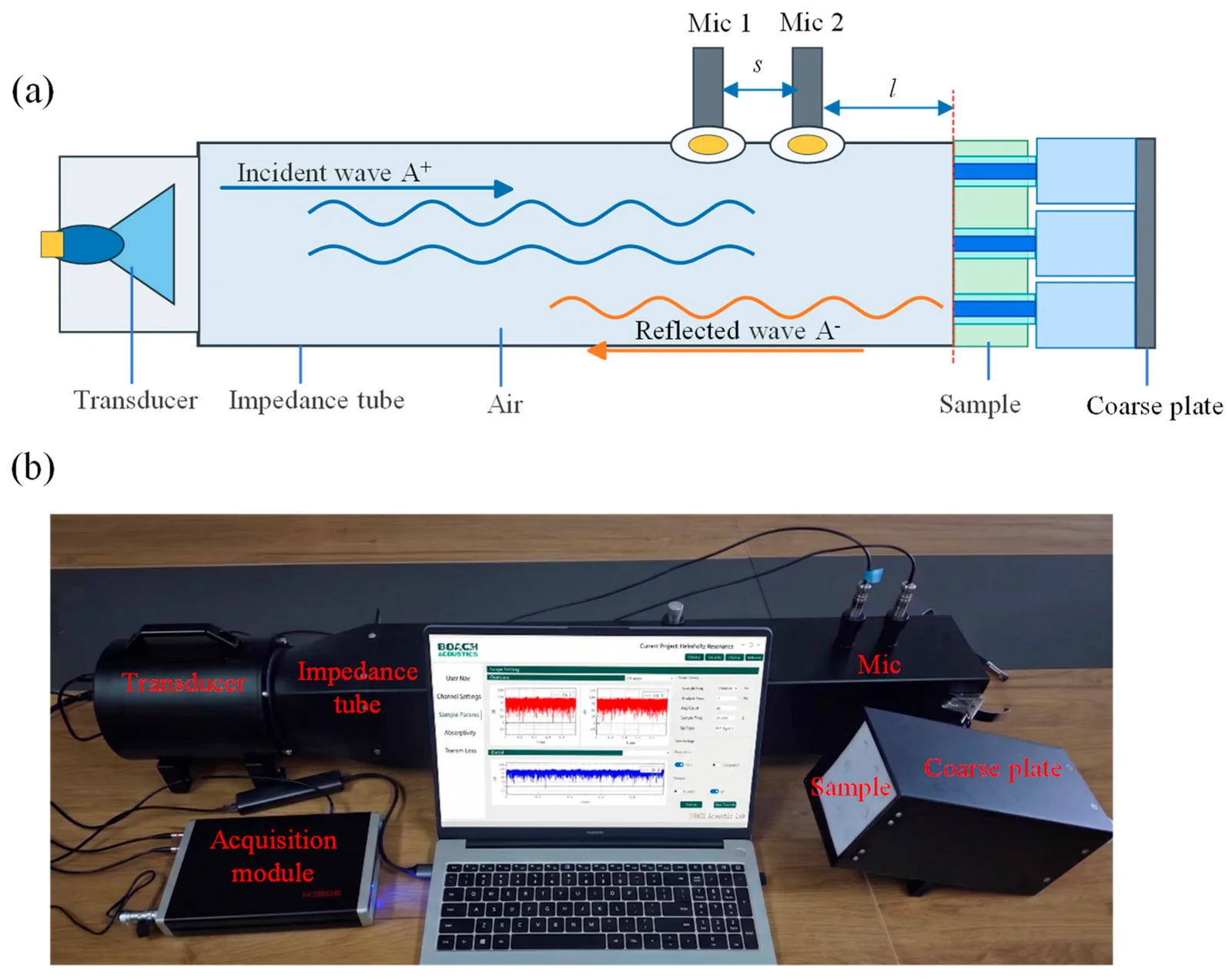
Acoustic impedance tube measurement: (**a**) schematic diagram of the testing principle; (**b**) experimental setup.

To evaluate the ability of the proposed theoretical model to predict the sound absorption performance of the material, normal-incidence absorption tests are conducted using a BOACH square impedance tube (Suzhou Leopard Acoustic Technology Co., Ltd., Suzhou China) with an internal cross-section of 100 × 100 mm. The measurement principle and experimental setup are shown in Figure 10a and Figure 10b, respectively. The tests followed the two-microphone transfer function method specified in ISO 10534-2 [51]. Mic 2 is positioned closer to the specimen than Mic 1. The microphone spacing is *s*, and the distance between Mic 2 and the specimen surface is *l*. A broadband excitation signal is generated by the computer, transmitted to the front-end unit, amplified by the power amplifier, and used to drive the loudspeaker, thereby producing a plane wave inside the impedance tube. The acoustic signals are acquired using a DRH08 (Suzhou Leopard Acoustic Technology Co., Ltd., Suzhou, China) data acquisition card at a sampling frequency of 25,600 Hz. The frequency resolution is 1 Hz, and 60 averages are used for each measurement. The experiments are conducted at an ambient temperature of approximately 25 °C and an atmospheric pressure of *P*_0_. The acquired signals are converted into digital data, from which the sound absorption coefficient is calculated and displayed in real time. Because the plane-wave cut-off frequency is approximately *f* = 0.5*c*_0_/*d* = 1700 Hz, the valid measurement range is set to 340–1700 Hz. The fabricated specimen has an external footprint of 99.5 × 99.5 mm, allowing a nominal 0.5 mm assembly clearance inside the square tube. During testing, the specimen is mounted at the tube termination against a rigid backing plate, and the remaining clearance is completely filled with sealing tape to minimize edge leakage.

When the measurement frequency is below the cut-off frequency of the higher-order tube modes, the sound field inside the impedance tube can be approximated as a one-dimensional plane wave. Taking the sample surface as *z* = 0, the acoustic pressure in the tube is written as the superposition of the incident and reflected waves:

(30) $$p(z)=A^{+}e^{jk_{0}z}+A^{-}e^{-jk_{0}z}$$

where *A*^+^ and *A*^−^ are the amplitudes of the incident and reflected waves, respectively, and *k*_0_ = *w*/*c*_0_ is the wavenumber in air. The reflection coefficient at the sample surface is defined as:

(31) $$R=\frac{A^{-}}{A^{+}}$$

In the two-Mic transfer function method, the complex pressure spectra *p*_1_ and *p*_2_ measured at Mic 1 and 2 are used to determine the transfer function:

(32) $$H_{12}=\frac{p_{2}}{p_{1}}$$

Here, *s* = 50 mm is the microphone spacing, and *l* = 230 mm is the distance between Mic 2 and the sample surface. The reflection coefficient at the sample surface is then obtained as:

(33) $$R=\left[\frac{H_{12}-e^{-jk_{0}s}}{e^{jk_{0}s}-H_{12}}\right]e^{2jk_{0}(l+s)}$$

The normal-incidence absorption coefficient and surface impedance are subsequently calculated as:

(34) $$\alpha =1-{\left|R\right|}^{2},\;Z_{s}=Z_{0}\frac{1+R}{1-R}$$

**Figure 11:**
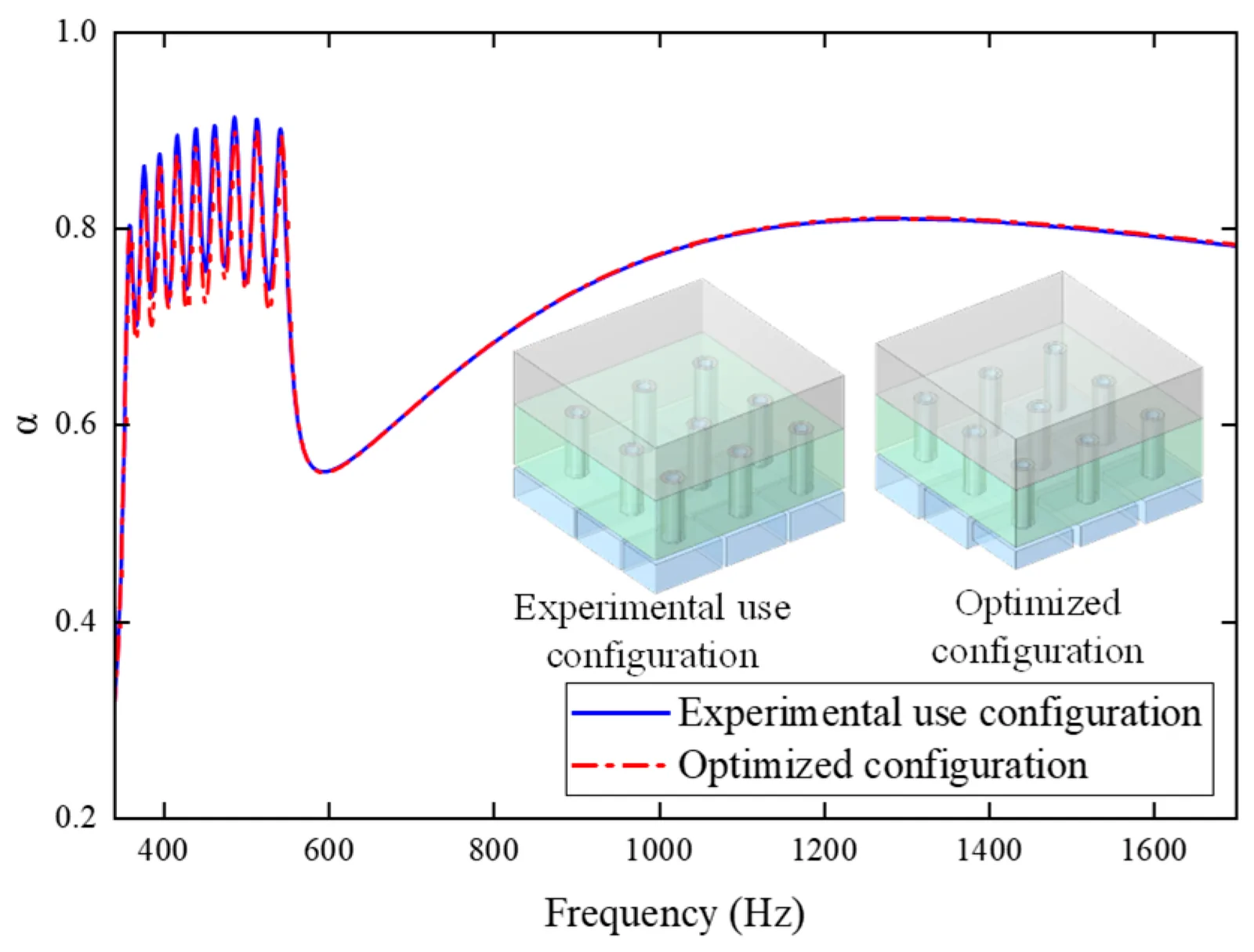
Validation of the geometry conversion from the optimized variable-depth design to the fabricated common-height specimen.

The optimization in Section 3 produces nine target back-cavity volumes. Direct realization of these volumes using identical in-plane cell areas and different cavity depths would make the largest cavity determine the total specimen thickness, leaving unused space behind the shallower cavities. The nonuniform rear surfaces would also hinder uniform contact with the rigid backing plate, and residual gaps could introduce additional compliance or leakage-related errors. Therefore, before fabrication, the original optimized geometry and the proposed common-height, area-adjusted geometry are compared by simulations under otherwise identical conditions. The original optimized geometry uses equal in-plane cell areas and different back-cavity depths, whereas the proposed geometry uses adjusted in-plane cavity areas and a common back-cavity height of *h_c_* = 13.36 mm. As shown in Figure 11, the two absorption responses are highly consistent. The RMSE is 0.0065 over 50–2000 Hz. This result confirms that the geometry conversion introduces only a small response difference and preserves the designed low-frequency absorption behavior while improving specimen compactness and mounting reliability.

**Figure 12:**
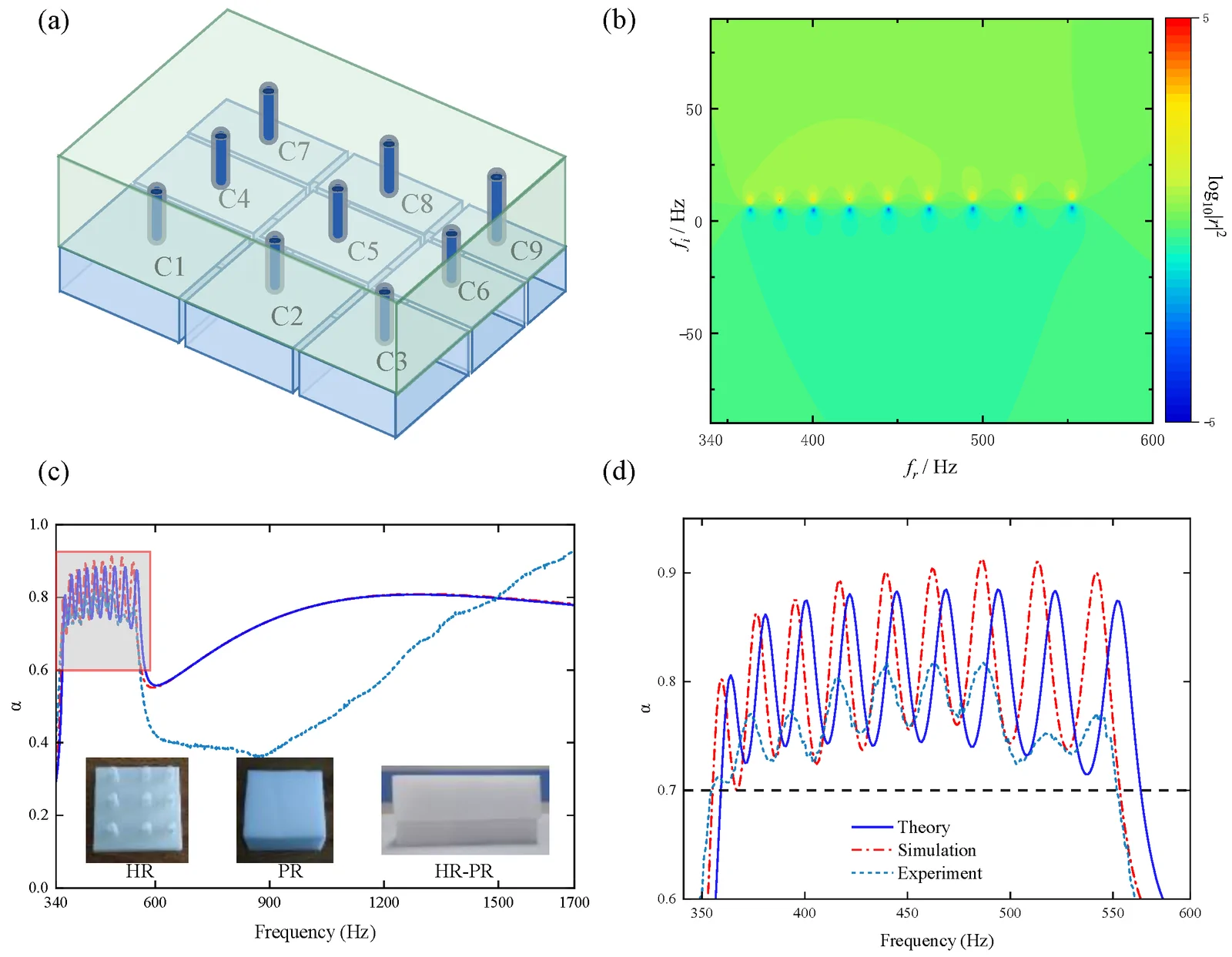
Experimental validation of the optimized HR–PR material: (**a**) geometry of the optimized sample; (**b**) zero-pole distribution; (**c**) theoretical, simulated, and experimental absorption coefficients; (**d**) enlarged view from 340 to 600 Hz.

Based on this validated geometry, the 3 × 3 specimen is fabricated by stereolithography (SLA) using a photopolymer resin. To allow insertion into the 100 × 100 mm BOACH square impedance tube, a nominal 0.5 mm assembly clearance is reserved on the specimen footprint, resulting in a fabricated external size of 99.5 × 99.5 mm. The projected back-cavity dimensions of C1–C9 are 33.72 mm × 40.28 mm, 30.55 mm × 40.42 mm, 27.23 mm × 40.50 mm, 33.74 mm × 29.68 mm, 30.40 mm × 29.62 mm, 27.36 mm × 29.63 mm, 33.83 mm × 21.54 mm, 30.42 mm × 21.46 mm, and 27.25 mm × 21.37 mm, respectively. A rigid spacing of 2 mm is kept between neighboring cavities. For *r_n_* = 3 mm and *l_n_* = 30 mm, the resulting total geometric thickness is about 43.36 mm. The corresponding geometry is shown in Figure 12a. A polyurethane foam layer with a thickness of 28 mm is inserted as the PR component, using the same material parameters as those adopted in the theoretical and numerical models and is supplied by Shanghai Zhenmo New Materials Co., Ltd, Shanghai, China. During testing, the specimen is mounted at the tube termination with the coarse plate and a rigid backing plate behind it, and the 0.5 mm assembly clearance is completely filled with sealing tape to suppress edge leakage.

Figure 12b shows that the zeros and poles are distributed over 340–600 Hz, with several staggered modal features close to the real-frequency axis. Reflection zeros near this axis improve impedance matching and generate absorption peaks, whereas the neighboring poles represent localized energy storage in the HR branches. This alternating zero-pole pattern enables the nine HR branches with different back-cavity volumes to form a coupled multi-peak low-frequency response. For the material in Figure 12a, Figure 12c compares the theoretical, simulated, and measured absorption coefficients, and Figure 12d enlarges the 340–600 Hz range. The experimental curve follows the theoretical and simulated trends in this low-frequency range. Quantitatively, in the 340–600 Hz range, the RMSE is 0.0931 between theory and experiment and 0.0722 between COMSOL and experiment. In the 360–563 Hz design interval, the corresponding RMSE values are 0.0776 and 0.0637, respectively. The measured *α* ≥ 0.7 band is 354–552 Hz, with a bandwidth of 198 Hz, close to the theoretical 360–563 Hz band with a bandwidth of 203 Hz. The remaining discrepancies, especially at higher frequencies, may be related to fabrication tolerances, porous material cutting and assembly errors, and possible acoustic leakage at the specimen tube interface despite tape sealing.

## 5. Conclusions

This study proposes an HR–PR composite absorber composed of cylindrical air necks, independent rigid back cavities, and a porous layer. The HR branches tune the low-frequency resonances, while the porous branch described by the JCA equivalent-fluid model provides mid-to-high-frequency viscous–thermal dissipation. Broadband low-frequency absorption is achieved by arranging nine different back-cavity volumes in parallel. Based on the neck-cavity equivalent-impedance model, the JCA porous-medium model, and the parallel surface impedance theory, a hierarchical theoretical model is established from the pure HR branch and pure PR branch to the nine-cell composite material. COMSOL acoustic simulations are then used to validate the theoretical predictions, and complex-frequency zero-pole analysis is employed to explain the formation mechanism of the low-frequency absorption peaks. Finally, the distribution of the nine HR peak frequencies is optimized to identify a broad continuous absorption band for *α* ≥ 0.7, and an air impedance tube experiment is used to verify the optimized material. The main conclusions are as follows:

1. A hierarchical theoretical model for the HR–PR composite absorber is established based on HR equivalent impedance, the JCA equivalent-fluid model, and parallel surface impedance theory. The model effectively predicts the low-frequency resonance peak positions within the current parameter range and reveals that broadband absorption originates from the synergy between low-frequency HR multi-peak coupling and mid-to-high-frequency PR viscous–thermal dissipation.
2. Complex-frequency zero-pole analysis shows that the low-frequency absorption peaks are closely related to reflection zeros located near the real-frequency axis. Nine different back-cavity volumes generate overlapped HR peaks in the low-frequency range, thereby raising the valley values between peaks and extending the continuous absorption band. The PR branch mainly provides dissipative compensation in the mid-to-high-frequency range. Therefore, the back-cavity volume distribution determines the low-frequency peak placement and continuous bandwidth, whereas the PR porous layer controls the mid-to-high-frequency platform and the damping level between peaks.
3. Under the *α* ≥ 0.7 criterion, the best-performing preset volume distribution provides a low-frequency continuous bandwidth of 154 Hz. After optimization, the continuous band satisfying *α* ≥ 0.7 is broadened to 360–563 Hz, corresponding to a bandwidth of 203 Hz. The optimized composite material agrees well with the impedance tube experimental result in the overall absorption trend.

In summary, this work demonstrates the low-frequency broadband absorption of the HR–PR composite under a rigid-frame JCA framework and normal-incidence conditions, but several limitations remain. The present model does not explicitly account for porous-frame elasticity, acoustic-structure coupling, or oblique-incidence and diffuse-field conditions, and the porous parameters are adopted as effective values from the prior literature rather than being directly linked to the microstructure. The selected 360–563 Hz band is used as a representative low-frequency design window rather than a fixed application-specific spectrum. For other practical noise control scenarios, the same peak placement strategy can be retargeted by adjusting the HR resonance distribution and porous layer parameters according to the measured noise spectrum and available installation thickness. Future work may therefore incorporate more complete equivalent-fluid formulations such as JCAL or JCAPL, together with microstructural characterization, oblique-incidence tests, and transmission-loss validation, to improve the physical completeness and engineering applicability of the design.

## Figures and Tables

**Table 1:** Preset back-cavity height distributions and their influence on absorption peaks.

Scheme	*h*_1_	*h*_2_	*h*_3_	*h*_4_	*h*_5_	*h*_6_	*h*_7_	*h*_8_	*h*_9_
Initial design	21.67	20.00	18.33	16.33	15.40	14.12	12.84	11.56	10.92
Uniform volume	21.67	20.33	18.98	17.64	16.30	14.95	13.61	12.26	10.92
Frequency spacing	21.67	19.61	17.84	16.29	14.94	13.75	12.69	11.76	10.92
Low frequency	21.67	21.26	20.28	18.93	17.36	15.69	14.01	12.41	10.92
High frequency	21.67	18.33	15.96	14.25	13.00	12.09	11.46	11.07	10.92

**Table 2:** Optimized back-cavity heights and resonance frequencies of the nine-unit cells.

Cell	1	2	3	4	5	6	7	8	9
*h_i_* (mm)	20.3	18.4	16.6	15.0	13.5	12.1	10.9	9.8	8.7
*f_i_* (Hz)	362.3	379.9	399.8	421.2	444.2	468.2	493.9	521.6	552.5

## Data Availability

The original contributions presented in this study are included in the article. Further inquiries can be directed to the corresponding authors.

## Abbreviations

The following abbreviations are used in this manuscript:

HR	Helmholtz resonator
PR	porous material
JCA	Johnson–Champoux–Allard model
